# The ergodicity solution of the cooperation puzzle

**DOI:** 10.1098/rsta.2020.0425

**Published:** 2022-07-11

**Authors:** Ole Peters, Alexander Adamou

**Affiliations:** ^1^ London Mathematical Laboratory, 8 Margravine Gardens, London W6 8RH, UK; ^2^ Santa Fe Institute, 1399 Hyde Park Road, Santa Fe, NM 87501, USA

**Keywords:** cooperation, ergodicity, evolution

## Abstract

When two entities cooperate by sharing resources, one relinquishes something of value to the other. This apparent altruism is frequently observed in nature. Why? Classical treatments assume circumstances where combining resources creates an immediate benefit, e.g. through complementarity or thresholds. Here we ask whether cooperation is predictable without such circumstances. We study a model in which resources self-multiply with fluctuations, a null model of a range of phenomena from viral spread to financial investment. Two fundamental growth rates exist: the ensemble-average growth rate, achieved by the average resources of a large population; and the time-average growth rate, achieved by individual resources over a long time. As a consequence of non-ergodicity, the latter is lower than the former by a term which depends on fluctuation size. Repeated pooling and sharing of resources reduces the effective size of fluctuations and increases the time-average growth rate, which approaches the ensemble-average growth rate in the many-cooperator limit. Therefore, cooperation is advantageous in our model for the simple reason that those who do it grow faster than those who do not. We offer this as a candidate explanation for observed cooperation in rudimentary environments, and as a behavioural baseline for cooperation more generally.

This article is part of the theme issue ‘Emergent phenomena in complex physical and socio-technical systems: from cells to societies’.

## 1. Introduction

They give that they may live, for to withhold is to perish [1].

Living beings exist not as minimal self-reproducing units but as cells, organisms, families, institutions, nations and so on. Cooperation, which we model as sharing resources, is ubiquitous in nature and society.

This ubiquity is puzzling because sharing seems *prima facie* to require the better-off member of a cooperating pair to relinquish something of value to the worse-off member, with nothing in return. If naked altruism is an unsatisfactory explanation of evolved behaviour, then we must expose the advantage derived by the better-off entity in such an arrangement.

Classical explanations involve two ideas. The first is that a net benefit arises when two entities cooperate. Specifically, the gain of the recipient—often expressed in terms of ‘fitness’—exceeds the cost to the donor. The second is that, over time, some of the net benefit finds its way back to the donor. This can happen through reciprocity, where past donors become future recipients, or through relatedness, where the recipient carries genetic material that the donor wants to propagate. Nowak [2] offers a comprehensive account of this approach, describing cooperative structures that are possible when such benefits exist. Aktipis [3] explores the regulatory mechanisms that facilitate such structures at large scales and over long times.

A net benefit typically requires complementarity in the shared resources, or a resource threshold below which a desired outcome is unachievable. Complementarity can arise through differences in resource types, such as the knowledge and materials required to send a human to the moon, or through splitting tasks into complementary parts, such as one hunter chasing prey towards the other. Thresholds are common in human economic activity, e.g. business partners combining their resources to purchase a productive asset, such as manufacturing equipment, which each could not afford alone. In all cases, attendant circumstances are such that the whole is deterministically ‘more than the sum of its parts’.

Here we ask whether cooperation can arise without such effects. We study this question theoretically in a model population whose members’ resources follow noisy multiplicative growth, i.e. periodic multiplication by a random factor. For tractability, we use geometric Brownian motion (GBM), a continuous-time stochastic process in which resource multipliers are normally distributed. GBM is the limiting case of a broad class of multiplicative growth processes. Its universality makes it suitable for modelling a wide range of phenomena, from the spread of a virus to the compounding of investments. The message of this study is general and we refer abstractly to ‘populations’, ‘entities’ and ‘resources’, allowing readers to decide what interests them specifically. Importantly, resources in our model are identical in all respects other than their random fluctuations. This allows us to study cooperation under simple conditions, where the effects invoked in classical explanations do not exist.

There are two fundamental growth rates in noisy multiplicative growth. The *ensemble-average growth rate* is that achieved by the population average in the large-population limit. It is independent of fluctuations. The *time-average growth rate* is that achieved by a single entity in the long-time limit. It is lower than the ensemble-average growth rate by a fluctuation-dependent term. The difference between these growth rates is a manifestation of non-ergodicity; see [4] and references therein. We find that repeated pooling and sharing reduces the net effect of fluctuations, thereby increasing the time-average growth rate of each cooperator’s resources, which approaches the ensemble-average growth rate as the number of cooperators increases. Therefore, cooperation in our model is advantageous for the simple reason that those who do it outgrow those who do not.

We suggest two major implications of this theoretical finding. Firstly, it provides a candidate explanation for observed cooperation in real settings too simple for classical explanations to hold. One tantalizing example is the transition from unicellular to multicellular life. Classical explanations usually involve the emergence of some new function, e.g. the ability of a multicellular organism to swim up a nutrient gradient [5,6]. However, a new function requires a degree of complexity absent in early unicellular life. A credible theory of evolution must explain not only the rich tapestry of cooperative structure we see today but also early cooperative steps—such as from single cell to cell pair—in rudimentary environments. Similar questions arise in the formation of early human societies and in human activities prior to advanced technologies, such as land management before the agricultural revolution [7].

Secondly, our finding establishes a leading-order result, or behavioural baseline, from which deviations can be studied. If, as we suggest, cooperation is beneficial by default in a model with minimal assumptions, then special explanations are required when it is not observed in nature. Moreover, by showing that cooperation has a quantifiable benefit to individuals who engage in it, this result counsels against overly narrow definitions of self-interest.

Our work relates closely to the biological literature on geometric mean fitness, on which the negative effects of fluctuations have long been recognized [8–11]. We speak of growth rates and not fitness, since they are physically measurable and unequivocally defined, whereas fitness has multiple definitions [11]. Among entities whose resources undergo noisy multiplicative growth, those with the highest time-average growth rates will dominate their environment over time.

Also related is the literature on bet-hedging, in which sharing or diversification mechanisms are employed to reduce the variance of resource yields, often at the cost of reducing their mean. Phenomena such as land scattering by peasant farmers [7], food sharing by foragers [12] and, more recently, helping behaviours of birds [13] are explained as variance-reduction strategies, with the aim of reducing the frequency of catastrophic shortfalls and prolonging survival. Uitdehaag [14] explores a model similar to ours, in which multiplicative growers share alternately their surpluses under a no-net-donor constraint. He finds that fluctuation-induced reductions in proliferative success are best countered by sharing between entities with anticorrelated fluctuations, proposing this as an explanation of mutualism between specialists. Starrfelt & Kokko [15] formalize bet-hedging as a trade-off between means, variances and correlations of resource increments. Schreiber [16] relates this to the probability of bet-hedger genotypes dominating a population over time, while Kennedy *et al.* [17] express it as a special case of a general model of cooperation which allows for relatedness between entities [18].

We present our work as follows. In §2, we introduce our model of noisy multiplicative growth and review its properties. In §3, we describe our cooperation protocol in which entities grow, pool and share their resources repeatedly. In §4, we find that cooperation increases the time-average growth rate of resources of similar entities and we discuss whether this constitutes a type of group selection. In §5, we discuss generalizations of our model to dissimilar entities and correlated fluctuations. Finally, we summarize our findings in §6.

## 2. Noisy multiplicative growth

Here we introduce our underlying model of resource growth in the absence of cooperation, to which we add a resource-sharing protocol in §3.

Let $x_{i}(t)$ be the resources of entity i at time t. We assume each $x_{i}(t)$ follows noisy multiplicative growth, meaning that it is multiplied periodically by realizations of a random variable. While we speak abstractly of the resources of an entity, our analysis is agnostic to replacement of ‘resources’ by ‘biomass’, ‘food’, ‘wealth’ and so on, and of ‘entity’ by ‘cell’, ‘organism’, ‘colony’, ‘person’, ‘tribe’ and so on. Cooperation occurs in many domains and at many scales.

Our specific model is GBM, where the change in resources over a short time step is a normally distributed random multiple of the existing resources. More formally, $x_{i}(t)$ follows the Itô stochastic differential equation,

2.1 $$dx_{i}(t)=x_{i}(t)\;(\mu \;dt+\sigma \;dW_{i}(t)),$$

where μ is the drift and σ is the volatility. The $dW_{i}(t)$ are independent and identically distributed increments of the Wiener process which are normal variates with zero mean and variance dt.

The realizations $dW_{i}(t)$ in equation (2.1) are indexed by both i and t. This is because we anticipate different fluctuations for different entities and at different times, for example due to environmental conditions that vary over space and time. This spatio-temporal distinction is important in the biological literature, where within-generation and between-generation variances of outcomes are studied [15,16]. Furthermore, we assume the $dW_{i}(t)$ are independent. We explore the effects of correlated fluctuations, i.e. of entities experiencing consistently similar or dissimilar conditions, in §5.

GBM is a universal model because it is an attractor for more complex models of multiplicative growth. Since its logarithmic increments are normally distributed, any stochastic process to whose logarithmic increments the central limit theorem applies will be distributed as a GBM in the many-increment limit. Formal treatments can be found in [19,20]. The key point is that there exists a broad class of multiplicative processes which, over time scales much longer than a single time step, are well approximated by GBM.

We note that GBM is a model of unconstrained self-reproduction. Growth that is limited by resource or space constraints or by predation would be poorly described by equation (2.1). The water lily of the famous riddle, told in [21], stops growing exponentially once it covers the pond.

It is an established property of GBM, which we quote without derivation, that its solution follows a time-dependent lognormal distribution,

2.2 $$\ln x_{i}(t)∼N\left(\ln x_{i}(0)+\left(\mu -\frac{\sigma^{2}}{2}\right)t,\sigma^{2}t\right),$$

where $N(m,s^{2})$ denotes the normal distribution with mean *m* and variance *s*^2^. Therefore, over a time period T, each entity’s resources experience a random exponential growth rate,

2.3 $$g(x_{i},T)\equiv \frac{1}{T}\ln\left(\frac{x_{i}(T)}{x_{i}(0)}\right),$$

which follows a normal distribution,

2.4 $$g(x_{i},T)∼N\left(\mu -\frac{\sigma^{2}}{2},\frac{\sigma^{2}}{T}\right).$$

Imagine starting many cell cultures in separate Petri dishes and watching their biomasses evolve according to equation (2.1) for time T. Assume the dishes are large enough and T is short enough that growth does not slow for want of agar. Equation (2.4) says that the observed growth rates would be normally distributed around $\mu -\sigma^{2}/2$, with time-decaying variance $\sigma^{2}/T$.

The negative term $-\sigma^{2}/2$ may be surprising, given that the multiplicative changes in equation (2.1) are distributed symmetrically about μ dt. It is, however, a well-known property, usually derived using Itô calculus as in [22]. In simple terms, it reflects the fact that the product of the symmetric perturbations of a number is less than its square, e.g. $(1+\epsilon )(1-\epsilon )=1-\epsilon^{2}<1$.

The ensemble average (or expectation value) of resources is defined as

2.5 $$\langle x(t)\rangle \equiv \lim_{N\to \infty }\frac{1}{N}\sum_{i=1}^{N}x_{i}(t).$$

Its evolution is computed by applying this operation to equation (2.1) to get

2.6 d⟨x(t)⟩=μ⟨x(t)⟩ dt,

since $\left\langle dW_{i}(t)\right\rangle =0$. With initial condition $x_{i}(0)=1$ for all i, this describes exponential growth, ⟨x(t)⟩=exp⁡(μt), at a rate equal to the drift, μ. We will call this the *ensemble-average growth rate* and denote it by

2.7 g(⟨x⟩)=μ.

The physical interpretation of this quantity is worth making explicit: it is the growth rate of the average resources of a population in the large-population limit.

One might guess that the growth rate observed in an individual trajectory will converge to equation (2.7) over time, but this would be a common conceptual error. Instead, the non-ergodicity of equation (2.1) manifests itself such that the growth rate observed in a single trajectory converges to a different value, called the *time-average growth rate*. This is the T→∞ limit of equation (2.4), in which the variance decays almost surely to zero to leave

2.8 $$\bar{g}(x_{i})\equiv \lim_{T\to \infty }g(x_{i},T)=\mu -\frac{\sigma^{2}}{2}.$$

We see in nature and society what has survived. In our model, the entity with the highest time-average growth rate will, regardless of its ensemble-average growth rate, come to dominate the environment’s resources in the long-time limit. The ratio of its resources to those of other entities will grow exponentially. Strategies which increase $\bar{g}(x_{i})$, regardless of their effect on g(⟨x⟩), will confer an evolutionary advantage on their adherents. We find that cooperation, specifically the repeated pooling and sharing of resources, is one such strategy.

## 3. Cooperation protocol

Having established the properties of equation (2.1), we introduce our model of cooperation. We start with a population of N non-cooperating entities, whose resources follow GBM with identical drift and volatility, i.e. equation (2.1) with i=1,…,N.

For simplicity, we consider a discrete version of equation (2.1). The non-cooperators’ resources grow over a finite time step, Δt, according to

3.1 $$\Delta x_{i}(t)=x_{i}(t)\left(\mu \Delta t+\sigma \xi_{i}(t)\sqrt{\Delta t}\right)$$

and

3.2 $$x_{i}(t+\Delta t)=x_{i}(t)+\Delta x_{i}(t),$$

where $\xi_{i}(t)$ are independent standard normal variates, $\xi_{i}(t)∼N(0,1)$.

**Figure 1.**
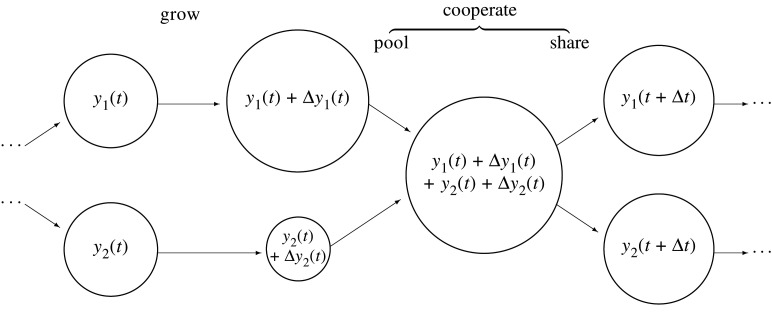
Cooperation dynamics. Two cooperators start each time step with equal resources. Then they grow independently according to equation (3.3). Then they cooperate by pooling resources and sharing them equally, according to equation (3.4). Then the next time step begins.

The cooperation mechanism, summarized pictorially for N=2 in figure 1, is as follows. Previously independent entities with resources $x_{i}(t)$ start to cooperate. This might happen through a genetic mutation which forces entities to share resources, as in the context of primitive life, or through more complex regulatory mechanisms, as in animal and human interactions. We label the resources of the cooperating entities $y_{i}(t)$ to distinguish them from the corresponding non-cooperators. We assume equal sharing of resources, $y_{i}(t)=y^{\left(N\right)}(t)$ for all i, where $y^{\left(N\right)}(t)$ denotes the per-entity resources for N equal cooperators.

In the discrete-time picture, each time step involves two phases. First, there is a growth phase, analogous to equation (3.1), in which each cooperator increases its resources by

3.3 $$\Delta y_{i}(t)=y^{\left(N\right)}(t)\left(\mu \Delta t+\sigma \xi_{i}(t)\sqrt{\Delta t}\right).$$

This is followed by a cooperation phase, replacing equation (3.2), in which resources are pooled and shared equally among the cooperators:

3.4 $$y^{\left(N\right)}(t+\Delta t)=\frac{1}{N}\sum_{i=1}^{N}(y^{\left(N\right)}(t)+\Delta y_{i}(t))=y^{\left(N\right)}(t)+\frac{1}{N}\sum_{i=1}^{N}\Delta y_{i}(t).$$

Equation (3.4) is equivalent to equal sharing of the sum of the individual fluctuations,

3.5 $$\Delta y^{\left(N\right)}(t)=\frac{1}{N}\sum_{i=1}^{N}\Delta y_{i}(t).$$

Our model of cooperation is necessarily technical. A lay version, presented as an interactive fable about farmers who boost their grain yields by repeated pooling and sharing, is available at www.farmersfable.org.

Cooperation has no direct cost in this protocol. In reality, sharing often requires a coordinating mechanism. For example, large organisms have circulatory systems to disseminate nutrients, and human societies have administrative systems to redistribute resources. Such mechanisms may have costs that make cooperation disadvantageous [3]. Equally, we ascribe no direct benefit to cooperation. Costs and benefits emerge as the effects of cooperation on time-average growth rates.

Nor do we consider the cheating problem or the walk-away option [3], although we will discuss later when breaking away will be beneficial. For now, our cooperators are unable to break their pact.

Substituting equation (3.3) into equation (3.5) yields the dynamic followed by the resources of each cooperator

3.6 $$\Delta y^{\left(N\right)}(t)=y^{\left(N\right)}(t)\left(\mu \Delta t+\frac{\sigma }{N}\sum_{i=1}^{N}\xi_{i}(t)\sqrt{\Delta t}\right).$$

The simplification of the final term leads to the key insight of this paper. Sums of independent normal variates are normal, so we can define a single standard normal variate,

3.7 $$\xi^{\left(N\right)}(t)\equiv \frac{1}{\sqrt{N}}\sum_{i=1}^{N}\xi_{i}(t)∼N(0,1),$$

and rewrite equation (3.6) as

3.8 $$\Delta y^{\left(N\right)}(t)=y^{\left(N\right)}(t)\left(\mu \Delta t+\frac{\sigma }{\sqrt{N}}\;\xi^{\left(N\right)}(t)\sqrt{\Delta t}\right).$$

Thus, the net effect of N individual fluctuations pooled and shared is a single equivalent fluctuation, whose amplitude is $1/\sqrt{N}$ times the amplitude of the individual fluctuations. Substituting into equation (3.6) and letting the time increment Δt→0, we recover a stochastic differential equation of the same form as equation (2.1) and with the same drift μ, but with the volatility reduced from σ to $\sigma /\sqrt{N}$,

3.9 $$dy^{\left(N\right)}(t)=y^{\left(N\right)}(t)\left(\mu \;dt+\frac{\sigma }{\sqrt{N}}\;dW^{\left(N\right)}(t)\right).$$

The effect of this volatility reduction on the time-average growth rate of resources provides our answer to the cooperation puzzle, as we shall now show.

## 4. Ergodicity solution of the cooperation puzzle

The ensemble-average growth rates of $x_{i}(t)$ under equation (2.1) and $y^{\left(N\right)}(t)$ under equation (3.9) are identical: μ. From this perspective, there is no incentive to cooperate. Moreover, an entity that realizes a large positive resource change during a growth phase, equation (3.3), could keep its fortuitous gain by breaking the cooperative pact, without altering its ensemble-average growth rate. Thus, analysing the growth of the ensemble average of resources gives no reason for cooperation to arise and, if it does arise, a good reason for it to end. From this perspective, cooperation looks fragile at best and its frequent observation in nature seems puzzling.

The solution of the puzzle requires a change in perspective. The ensemble-average growth rate is uninformative of the growth of a single entity (or, indeed, any finite cooperative) over time. From the perspective of an individual, it is the growth rate achieved by averaging over infinitely many parallel realizations of its resources—an irrelevant fiction. It is more realistic to assume that an individual cares about its time-average growth rate, which better approximates what it will actually achieve in one resource trajectory over time.

We know from equation (2.8) that the time-average growth rate for non-cooperating entities is $\bar{g}(x_{i})=\mu -\sigma^{2}/2$. Under cooperative dynamics, equation (3.9), the volatility decreases from σ to $\sigma /\sqrt{N}$, which when substituted into equation (2.8) yields a higher time-average growth rate,

4.1 $$\bar{g}(y^{\left(N\right)})=\mu -\frac{\sigma^{2}}{2N}.$$

So, in the presence of fluctuations, σ>0, cooperators grow faster than non-cooperators in the long run. The growth rate premium increases with the number of cooperators as 1−1/N,

4.2 $$\bar{g}(y^{\left(N\right)})-\bar{g}(x_{i})=\frac{\sigma^{2}}{2}\left(1-\frac{1}{N}\right),$$

implying that larger cooperatives are favoured over smaller ones. This premium increases most rapidly when N is small—from single entities, to pairs, to triplets. As the cooperative expands, the benefit gained by adding each new member diminishes [12].

**Figure 2.**
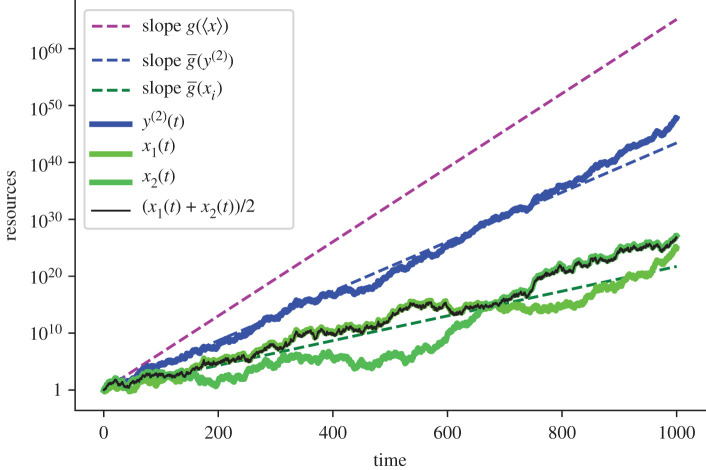
Typical trajectories for two non-cooperating (green) entities and for the corresponding cooperating entities (blue) on a logarithmic resource scale. Over time *t*, the volatility reduction achieved by the cooperators leads to faster growth. The thin black line shows the average resources of the non-cooperators, which is far inferior to those of the cooperators. In a literal mathematical sense, the whole, $y_{1}(t)+y_{2}(t)$, is more than the sum of its parts, $x_{1}(t)+x_{2}(t)$. Resource growth at the ensemble-average growth rate (pink) is approached in the limit of infinitely many cooperators. Parameters: μ=0.15 per time unit, $\sigma^{2}=0.2$ per time unit. The discrete time step Δt was set to 0.1 time units. The code to generate the figure is in the electronic supplementary material, cooperate.py. (Online version in colour.)

In our model, cooperators will eventually dominate the environment and cooperation will become ubiquitous. The effect is illustrated in figure 2 by direct simulation of equations (3.1), (3.2) and (3.6). It constitutes our main result. We see that over a long enough time, the resources of each member of a cooperating pair (blue) grow faster than the resources of the corresponding non-cooperators (green). They also grow faster than the average resources of the non-cooperators (black), showing that cooperation and averaging are not equivalent operations.

In equation (4.1), we see that the time-average growth rate of a cooperator approaches the ensemble-average growth rate of a non-cooperator as the number of cooperators grows large,

4.3 $$\lim_{N\to \infty }\bar{g}(y^{\left(N\right)})=\mu .$$

The pink dashed line in figure 2 plots resource growth at the ensemble-average growth rate. This is relevant to members of large cooperatives, equation (4.3), but bears little resemblance to that of the resource growth of non-cooperators (green) and members of small cooperatives (blue). This is the essence of the ergodicity debate: features emerging from fluctuations when averaging over a statistical ensemble do not, in general, also emerge in individual systems over time.

### (a) The group selection debate

One explanation of observed cooperative behaviour—known as group or multi-level selection—has led to controversy among evolutionary biologists; see [23] and references therein. While it is unclear whether the debate is semantic or substantive [24], we nevertheless offer a brief summary of it and its relation to our work.

The dominant paradigm in evolutionary biology, originating in [25], is that natural selection acts at the level of the replicating unit, such as an organism or its genotype. We call this individual selection to distinguish it from group selection. Put simply, a trait which increases the reproductive success of individuals over those with other traits will tend to increase its prevalence in a population over time.

The consensus view is that individual selection explains observed cooperation [26]. The premise is that all cooperative behaviour is beneficial to the individual engaging in it. If such behaviour is interpreted as *prima facie* disadvantageous, this is simply a mistake arising from an over-narrow view of the behaviour’s consequences or the individual’s interests. Reciprocity and relatedness are examples where apparently costly aid is actually self-interested: either because it leads to receipt of aid in the future, or because it benefits the genotype of the aid-giver. Indeed, the cooperation puzzle presented here may be viewed as an artefact of ignoring the wider circumstances of a resource-sharing act, namely that it belongs to a sequence of similar acts.

Group selection is an alternative paradigm in which natural selection occurs at group level [27–29]. Behaviour which is apparently costly to the individual is rendered unproblematic, since it is the group to which selection pressure applies. Thus, individual traits can evolve which are selectively good for the group but selectively bad for individuals within the group. This approach is controversial *inter alia* because groups are not considered replicating units and because individual selection is already deemed sufficient to explain cooperation. We offer no new analysis of this controversy, which is beyond the scope of this paper.

However, we suggest that our model of cooperation sits naturally in the individual-selection paradigm. Individuals pool and share resources because, over a temporal sequence of interactions, it is individually advantageous for them to do so. Behavioural traits are good for the group precisely because they are good for every member of the group. Group selection is not needed because the interests of the group and its members are fully aligned.

This raises the question of whether group and individual interests could become misaligned in our model. For example, could invasion of a resource-sharing cooperative by exploitative non-cooperators create a situation where cooperators engage in personally costly behaviour to benefit the group or a subset thereof?

Leaving aside the question of whether such a mixed group would be sustainable, which would depend on the exploitation mechanism, it is clear that breaking the cooperative pact once creates a short-term advantage for the pact-breaker. However, provided regulation exists to prohibit repeated pact-breaking, this will not translate into a long-term advantage. One simple way in which cooperators could protect themselves from repeated exploitation would be to recognize past exploiters and refuse to interact with them in future—an ‘ignore-for-tat’ policy.

The penalty for non-cooperation would be ejection from the cooperative, reduction of the time-average growth rate from $\mu -\sigma^{2}/(2N)$ to $\mu -\sigma^{2}/2$ and the eventual exponential irrelevance this implies. Detection and punishment need not be perfect. Even if the penalty were exacted with non-zero probability at each betrayal, it would be only a matter of time before the traitor was expelled from the group and consigned to lag behind. However, since our main aim is to study *why* resource-sharing has an evolutionary advantage, rather than *how* it is enforced, we refrain from further development of this point.

## 5. Generalizations

### (a) Idiosyncratic entities

Real cooperatives have members of differing abilities as well as differing fortunes. The latter we model already as different realizations of the Wiener increments in equation (2.1). The former we can treat by generalizing equation (2.1) so that the entities have idiosyncratic drifts and volatilities,

5.1 $$dx_{i}(t)=x_{i}(t)(\mu_{i}\;dt+\sigma_{i}\;dW_{i}(t))$$

for i=1,…,N. The time-average growth rates, $\bar{g}(x_{i})=\mu_{i}-\sigma_{i}^{2}/2$, are now idiosyncratic. Some entities will, if left to their own devices, grow their resources faster than others. This raises questions. Does it benefit leaders to share with laggards? When should a non-cooperator join a cooperating group? When should the group allow it?

Repeating the analysis of growth, pooling and sharing yields a modified dynamic

5.2 $$\begin{array}{rl} \Delta y^{\left(N\right)}(t) & \;=y^{\left(N\right)}(t)\left(\frac{1}{N}\sum_{i=1}^{N}\mu_{i}\Delta t+\frac{1}{N}\sum_{i=1}^{N}\sigma_{i}\xi_{i}(t)\sqrt{\Delta t}\right)\\ & \;=y^{\left(N\right)}(t)\left(\mu^{\left(N\right)}\Delta t+\sigma^{\left(N\right)}(t)\xi^{\left(N\right)}\sqrt{\Delta t}\right), \end{array}$$

where $\xi^{\left(N\right)}(t)$ is a standard normal variate, as before, and

5.3 $$\mu^{\left(N\right)}\equiv \frac{1}{N}\sum_{i=1}^{N}\mu_{i},\;\sigma^{\left(N\right)}\equiv \frac{1}{N}\sqrt{\sum_{i=1}^{N}\sigma_{i}^{2}}$$

are the effective drift and volatility parameters. Therefore, the resources of the cooperators evolve according to

5.4 $$dy^{\left(N\right)}(t)=y^{\left(N\right)}(t)(\mu^{\left(N\right)}dt+\sigma^{\left(N\right)}dW^{\left(N\right)}(t)),$$

with time-average growth rate

5.5 $$\bar{g}(y^{\left(N\right)})=\mu^{\left(N\right)}-\frac{{\left(\sigma^{\left(N\right)}\right)}^{2}}{2}=\frac{1}{N}\sum_{i=1}^{N}\left(\mu_{i}-\frac{\sigma_{i}^{2}}{2N}\right).$$

This happens to be the sample mean of the time-average growth rates each entity would achieve in a cooperative of N like entities; cf. equation (4.1).

We can now answer the questions. It benefits entity j to be a member of the cooperative if $\bar{g}(y^{\left(N\right)})>\bar{g}(x_{j})$, i.e. if

5.6 $$\frac{1}{N}\sum_{i=1}^{N}\left(\mu_{i}-\frac{\sigma_{i}^{2}}{2N}\right)>\mu_{j}-\frac{\sigma_{j}^{2}}{2}.$$

Similarly, the cooperative benefits by having j as a member if

5.7 $$\frac{1}{N}\sum_{i=1}^{N}\left(\mu_{i}-\frac{\sigma_{i}^{2}}{2N}\right)>\frac{1}{N-1}\sum_{\begin{array}{c} i=1\\ i\neq j \end{array}}^{N}\left(\mu_{i}-\frac{\sigma_{i}^{2}}{2(N-1)}\right).$$

In reality, we may interpret this as saying that, even in simple set-ups, a better-skilled individual motivated by nothing but greed can still do better as part of society.

### (b) Correlated fluctuations

A second generalization concerns correlations. Fluctuations experienced by different entities are uncorrelated in our model. The $dW_{i}(t)$ in equation (2.1) and, consequently, the $\xi_{i}(t)$ in equation (3.1) onwards are independent random variables. In reality, cooperators are often spatially localized or socially connected, and they experience similar environmental or economic conditions at a given time. By allowing correlations in fluctuations across entities, i.e. correlated $\xi_{i}(t)$ and $\xi_{j}(t)$, our model can be adapted to describe such situations.

Suppose the $\xi_{i}(t)∼N(0,1)$ realized in a given time step, from t to t+Δt, are jointly normal and cross-correlated such that $\left\langle \xi_{i}(t)\xi_{j}(t)\right\rangle =\rho_{ij}$. Assume for simplicity that

5.8 $$\rho_{ij}=\left\{ \begin{array}{ll} 1, & i=j,\\ \rho , & i\neq j, \end{array}\right.$$

so that the fluctuations for all pairs of different entities have the same covariance, ρ, where −1≤ρ≤1. The more general case of a covariance matrix with unequal off-diagonal elements is also tractable, but adds complexity without illumination.

The presence of cross-correlations alters the evaluation of the sum of the normal variates in equation (3.6). We have now

5.9 $$\sum_{i=1}^{N}\xi_{i}(t)∼N(0,N+N(N-1)\rho ).$$

Positive variance requires ρ to be confined to −1/(N−1)≤ρ≤1 (meaning that perfect anticorrelation can exist only for N=2 and that anticorrelation is impossible as N→∞). Equation (5.9) suggests defining, analogously to equation (3.7), a standard normal variate

5.10 $$\xi^{\left(N\right)}(t)\equiv \frac{1}{\sqrt{N+N(N-1)\rho }}\sum_{i=1}^{N}\xi_{i}(t)∼N(0,1),$$

such that the change in $y^{\left(N\right)}(t)$ can be written as

5.11 $$\Delta y^{\left(N\right)}(t)=y^{\left(N\right)}(t)\left(\mu \Delta t+\sigma \sqrt{\frac{1+(N-1)\rho }{N}}\;\xi^{\left(N\right)}(t)\sqrt{\Delta t}\right),$$

analogous to equation (3.8) in the uncorrelated case.

Without correlations, cooperation reduces the amplitude of fluctuations from σ to $\sigma /\sqrt{N}$. With them, the fluctuation amplitude becomes

5.12 $$\sigma_{\text{corr}}\equiv \sigma \sqrt{\frac{1+(N-1)\rho }{N}}.$$

McCloskey [7] obtains the same expression for the variance of the annual yield of peasant farmers with scattered plots, as does Winterhalder [12] for teams of foragers sharing food.

The variation of $\sigma_{\text{corr}}$ with ρ and N delineates the main features of this model. Firstly, as a consistency check, we note that $\sigma_{\text{corr}}\to \sigma /\sqrt{N}$ as ρ→0 for fixed N, recovering the uncorrelated result in the appropriate limit.

For all N>1, we have $0\leq \sigma_{\text{corr}}\leq \sigma$, with $\sigma_{\text{corr}}=\sigma$ if and only if ρ=1. In other words, provided fluctuations are not perfectly correlated, a cooperation benefit always exists. This makes intuitive sense. With perfect correlation, all the $\xi_{i}$ are identical and sharing achieves nothing. The cooperative is equivalent to a giant individual following a single trajectory of equation (2.1). As soon as some variation is introduced between the fluctuations of the entities, the cooperation mechanism can begin to mitigate the negative effects of fluctuations on resource growth.

Furthermore, in the N→∞ limit we have $\sigma_{\text{corr}}\to \sigma \sqrt{\rho }$ with ρ≥0. The maximum time-average growth rate achievable by adding cooperators is, therefore,

5.13 $$\lim_{N\to \infty }\bar{g}(y^{\left(N\right)})=\mu -\frac{\sigma^{2}\rho }{2};$$

cf. equation (4.3). This cannot exceed μ and decreases as ρ increases. Again, this is consistent with intuition: as fluctuations become more correlated, the variation between them diminishes and the scope for beneficial cooperation narrows.

In our set-up, cooperation is enhanced by diversity in individual outcomes. Uitdehaag [14] illustrates the benefit of specialization in mutualistic societies with an extreme case of two entities, N=2, with perfect anticorrelation, ρ=−1. Here, $\sigma_{\text{corr}}=0$ and the time-average growth rate, $\mu -{\sigma_{\text{corr}}}^{2}/2$, becomes the ensemble-average growth rate, μ, with no further cooperation required. Indeed, maintaining diversity in outcomes is the explanation for land scattering favoured by McCloskey [7], citing first-hand accounts of reasoning given by farmers engaging in the practice. Similarly, where cooperation imposes structures that lead to a loss in diversity, the cooperation benefit is diminished and can be completely eliminated.

### (c) Other generalizations

Many other generalizations are possible. Berman *et al.* [30] analyse partial cooperation, in which entities pool and share a fraction of their resources, resembling taxation and redistribution in human societies. Stojkoski *et al.* [31] consider cooperation on a network, where entities pool and share resources only with those to whom they are connected. Yaari & Solomon [32] find an evolutionary advantage of cooperation in a different multiplicative process, with only two possible outcomes (win or loss) at each step, and we anticipate that similar results will hold for more general resource processes, such as those described by the present authors in [33].

## 6. Discussion

The cooperation puzzle refers to the common observation of spontaneous cooperative behaviour which, at first sight, appears unattractive to one cooperator. Classical treatments resolve it under circumstances where cooperation creates an immediate benefit, for instance where resources are complementary or where thresholds exist that an entity cannot surpass alone.

We have presented a simple and universal model of resource growth and sharing where cooperation proves beneficial in the absence of such circumstances. The time-average growth rate of resources is reduced by fluctuations. Pooling and sharing, by decreasing the effective amplitude of fluctuations, increases the time-average growth rate. Thus, cooperation is advantageous in our model because those who do it grow faster than those who do not. The simplicity and generality of our model make this a candidate explanation for the existence of cooperation in a wide range of real settings, especially in simple environments where classical explanations are unlikely to hold.

This minimalism allows us to ask which of cooperation and non-cooperation is the behavioural baseline and, therefore, which should be considered puzzling when observed. Following our analysis, we expect cooperation to be attractive when resources are, broadly speaking, self-reproductive—a class of situations encompassing the biomass of bacteria and the wealth of nations. In such situations, the absence of cooperation requires special explanations, such as coordination costs, diversity suppression (e.g. ‘groupthink’) or skill differences among would-be cooperators that discourage the most skilled from participating.

The impact of fluctuation reduction on long-time growth suggests that risk management has a rarely recognized significance. Good risk management does not merely reduce the size of up- and down-swings. It also improves long-time performance. This tangible economic benefit is frequently overlooked. The coordination costs and inefficiencies of insurance, pension and taxation systems are often discussed, without passing mention of this fundamental benefit that, we suppose, had much to do with their emergence in the first place.

Cooperation has no apparent benefit when judged by its effect on the ensemble-average growth rate of resources. Only the time-average growth rate reveals the benefit we have described. This work forms part of a research programme known as ergodicity economics; see e.g. [4]. This programme uses the ergodicity problem as a lens through which it redevelops the dominant economic formalism. The development of ergodic theory in the twentieth century provided the concepts needed to analyse stochastic growth processes like equation (2.1). Such analysis suggests that our natural tendency to cooperate—expressed in our gut feeling and moral sentiment—is in harmony with a careful formal analysis.

## Data Availability

The data are provided in the electronic supplementary material [34]. Data displayed in figure 2 were generated randomly using the code cooperate.py in the electronic supplementary material.
